# Ocular Involvement in Disseminated Cryptococcosis in Advanced HIV Disease: A Case Report and Focused Literature Review

**DOI:** 10.3390/jof12100755

**Published:** 2026-10-09

**Authors:** Raffaele Ferri, Salvatore Giuseppe Rizzello, Giorgia Taraschi, Katia Falasca, Jacopo Vecchiet

**Affiliations:** Infectious Disease Unit, Azienda Sanitaria Locale Lanciano Vasto Chieti and Gabriele D’Annunzio University, 66013 Chieti, Italy

**Keywords:** *Cryptococcus neoformans*, cryptococcosis, HIV, cryptococcal meningitis, chorioretinitis, ocular cryptococcosis, disseminated cryptococcosis, opportunistic infections

## Abstract

Ocular involvement is an uncommon manifestation of disseminated cryptococcosis and represents a diagnostic challenge in people with advanced HIV disease, in whom multiple opportunistic infections may coexist. We report a 46-year-old man with advanced HIV disease (CD4+ T-cell count, 12 cells/mm^3^) and microbiologically confirmed *Cryptococcus neoformans* meningitis and cryptococcemia. Persistent photophobia prompted ophthalmological evaluation, which disclosed bilateral multifocal whitish-yellow chorioretinal lesions. Optical coherence tomography demonstrated focal photoreceptor disruption and choroidal abnormalities, with corresponding choroidal flow-void areas on OCT angiography. In the absence of direct ocular microbiological or histopathological confirmation, the findings were classified as presumptive cryptococcal chorioretinitis. Systemic antifungal therapy with liposomal amphotericin B and flucytosine, followed by fluconazole consolidation, was associated with progressive anatomical regression, while visual acuity remained preserved bilaterally. A focused review of published HIV-associated cases revealed marked heterogeneity in ocular manifestations and, importantly, in the level of etiological confirmation. This case highlights the need to distinguish ophthalmological complications of cryptococcal meningitis from direct intraocular infection and to differentiate presumptive ocular involvement from microbiologically or histopathologically confirmed disease. Integrating systemic microbiological findings, exclusion of competing opportunistic infections, ophthalmological assessment, and multimodal imaging may support more rigorous etiological attribution of ocular abnormalities in disseminated cryptococcosis.

## 1. Introduction

Cryptococcal meningitis remains one of the leading causes of morbidity and mortality among people living with advanced HIV infection despite substantial advances in antiretroviral therapy and antifungal management [1].

Although the global incidence of HIV-associated cryptococcal meningitis has declined over the past decade, mortality remains high, approaching 50% in low-resource settings [2].

While cryptococcosis most commonly involves the central nervous system and lungs, disseminated disease may affect multiple organ systems. In patients with advanced HIV-associated immunosuppression, *Cryptococcus neoformans* remains the predominant causative species and exhibits a marked neurotropism, accounting for the high frequency of central nervous system involvement [3].

Ocular abnormalities are frequently observed in patients with cryptococcal meningitis; however, these are most commonly related to raised intracranial pressure and include papilledema, visual loss, and cranial nerve palsies [4,5]. In contrast, direct ocular involvement secondary to cryptococcal dissemination is uncommon and may manifest as choroiditis, chorioretinitis, or, less frequently, endogenous endophthalmitis, with hematogenous spread through the highly vascularized choroidal circulation considered the most likely route, although extension from meningeal infection has also been proposed [6].

The diagnosis of ocular cryptococcosis may be particularly challenging in patients with advanced HIV disease. Visual involvement may range from subtle visual disturbances to severe and potentially irreversible visual loss, making careful ophthalmological assessment relevant in patients with cryptococcal meningitis [4]. Furthermore, retinal lesions in severely immunocompromised patients raise a broad differential diagnosis, with cytomegalovirus retinitis representing the most common opportunistic ocular infection in this population [7]. Consequently, cryptococcal chorioretinitis may not be immediately suspected, especially in the absence of significant visual impairment.

The literature describing direct ocular cryptococcosis is predominantly composed of case reports and small observational series. Moreover, the level of etiological confirmation varies considerably. In some reports, *Cryptococcus* has been demonstrated directly in ocular tissue or vitreous fluid, whereas in others the ocular diagnosis has been inferred from microbiologically proven systemic or CNS cryptococcosis associated with a compatible ophthalmological phenotype [6,8,9,10,11,12].

This distinction assumes particular importance in advanced HIV disease, in which multiple opportunistic infections may coexist. A diagnosis of cryptococcal meningitis or cryptococcemia therefore increases the plausibility of cryptococcal ocular involvement but does not establish *Cryptococcus* as the cause of every ocular abnormality.

We report a patient with advanced HIV disease, microbiologically confirmed cryptococcal meningitis, and cryptococcemia who developed bilateral multifocal chorioretinal lesions during antifungal treatment. The case is discussed within a focused infectious-disease review addressing the distinction between neuro-ophthalmological complications and direct intraocular disease, disseminated cryptococcosis, diagnostic certainty, competing opportunistic infections, treatment, and clinical outcomes.

## 2. Case Presentation

### 2.1. Anamnesis and Clinical Presentation

A 46-year-old man presented to the Emergency Department with a two-week history of fever (maximum temperature 38 °C), profuse sweating, asthenia, and diffuse myalgia and arthralgia. He had been diagnosed with HIV infection in 2006 and was followed at our institution for CDC stage C3 disease secondary to previous *Pneumocystis jirovecii* pneumonia. His antiretroviral regimen consisted of darunavir/cobicistat/emtricitabine/tenofovir alafenamide administered as a single-tablet regimen.

During the epidemiological assessment, the patient reported having spent several months in his country of origin (Peru), during which his antiretroviral therapy had been interrupted because of limited drug availability.

On admission, laboratory investigations revealed advanced HIV-associated immunosuppression, with a CD4+ T-cell count of 12 cells/mm^3^, a CD8+ T-cell count of 192 cells/mm^3^, and a CD4/CD8 ratio of 0.06. Plasma HIV-RNA was 2801 copies/mL, and genotypic resistance testing did not identify any resistance-associated mutations.

### 2.2. Diagnostic and Microbiological Procedure

Because of profound immunosuppression and concomitant photophobia, brain computed tomography was performed prior to lumbar puncture and demonstrated no findings contraindicating the procedure. Cerebrospinal fluid (CSF) analysis revealed clear fluid with hyperproteinorrachia (102.8 mg/dL), hypoglycorrhachia (24 mg/dL; serum glucose 96 mg/dL), and pleocytosis with neutrophilic predominance (128 cells/μL; neutrophils 65%). Microbiological examination of CSF identified *Cryptococcus neoformans*, while serum cryptococcal antigen testing was also positive, confirming the diagnosis of cryptococcal meningitis. Blood cultures additionally yielded *C. neoformans*; the amphotericin B MIC was below the established epidemiological cutoff value (ECV), indicating a wild-type phenotype without evidence of acquired resistance.

Microbiological investigations for competing opportunistic infections were negative, including *Toxoplasma gondii* PCR, CMV-DNA testing in both blood and CSF, syphilis serology, and *Mycobacterium tuberculosis* PCR on CSF.

No aqueous humor or vitreous specimen was obtained during the diagnostic work-up; therefore, direct intraocular microbiological confirmation was not available. Given the profound immunosuppression, this represented an important diagnostic limitation, since atypical or concomitant ocular opportunistic infections could not be excluded with absolute certainty despite the negative systemic and CSF investigations.

Brain magnetic resonance imaging (MRI) demonstrated multiple small rounded hyperintense lesions involving the bilateral basal ganglia and capsular regions on T2-weighted and FLAIR sequences, without diffusion restriction or contrast enhancement, resulting in a “soap-bubble” appearance compatible with cryptococcal involvement.

### 2.3. Treatment and Clinical Course

Induction antifungal therapy was initiated with intravenous liposomal amphotericin B (3 mg/kg once daily) and oral flucytosine (25 mg/kg four times daily), in accordance with recommended induction regimens. Flucytosine was discontinued after 8 days because of thrombocytopenia, and therapeutic drug monitoring was not performed, as this assay was not available at our center. Serial lumbar punctures were performed during the induction phase for CSF reassessment and therapeutic drainage (Table 1). Following completion of the amphotericin B-based induction phase and documentation of sterile CSF cultures, treatment was transitioned to high-dose oral fluconazole (800 mg daily) for consolidation therapy.

**Figure 1:**
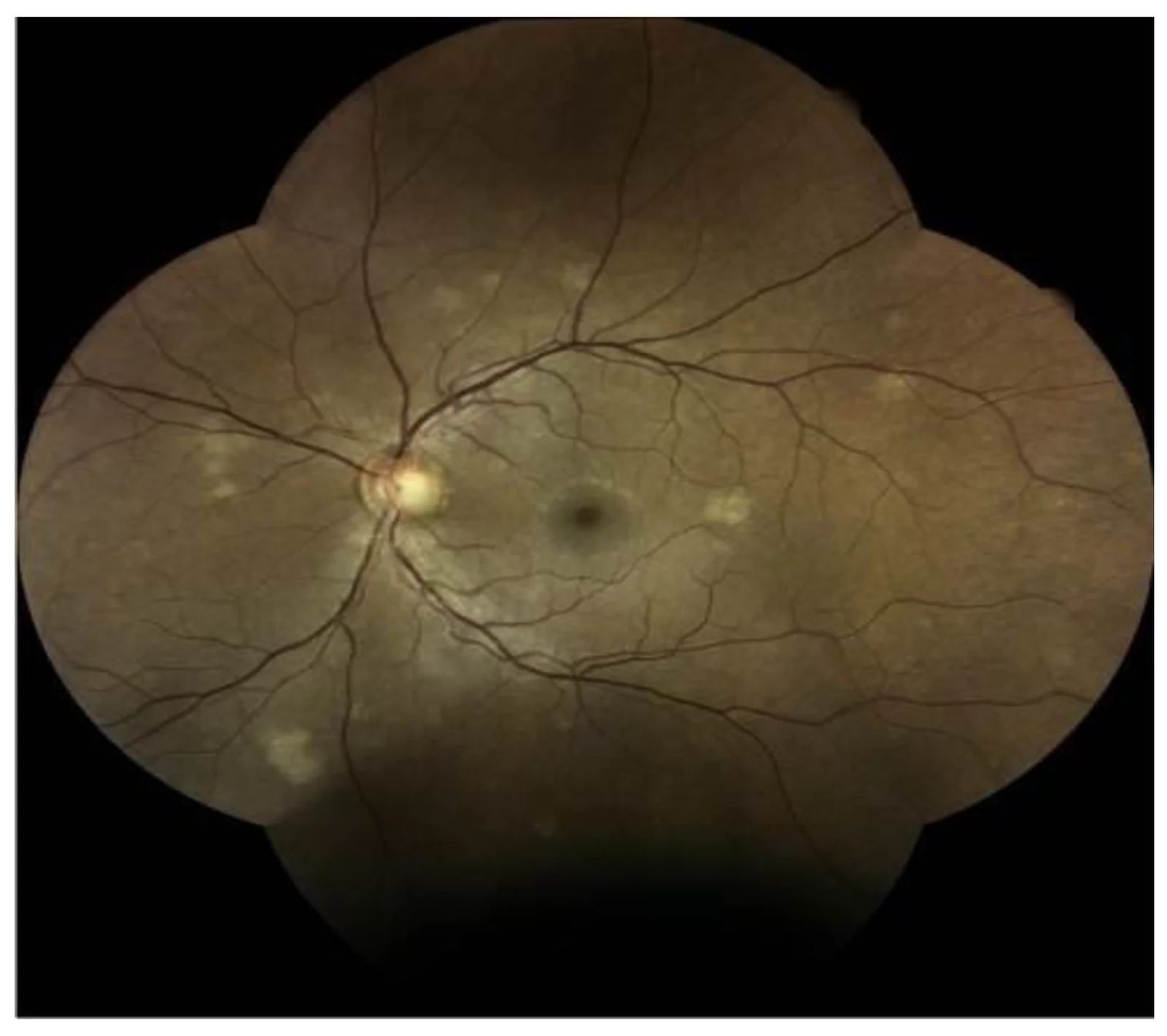
Fundus examination (EIDON, CenterVue S.p.A., Padua, Italy) revealed bilateral multifocal chorioretinitis with multiple whitish-yellow lesions involving the posterior pole and extending along the major retinal vascular arcades.

**Figure 2:**
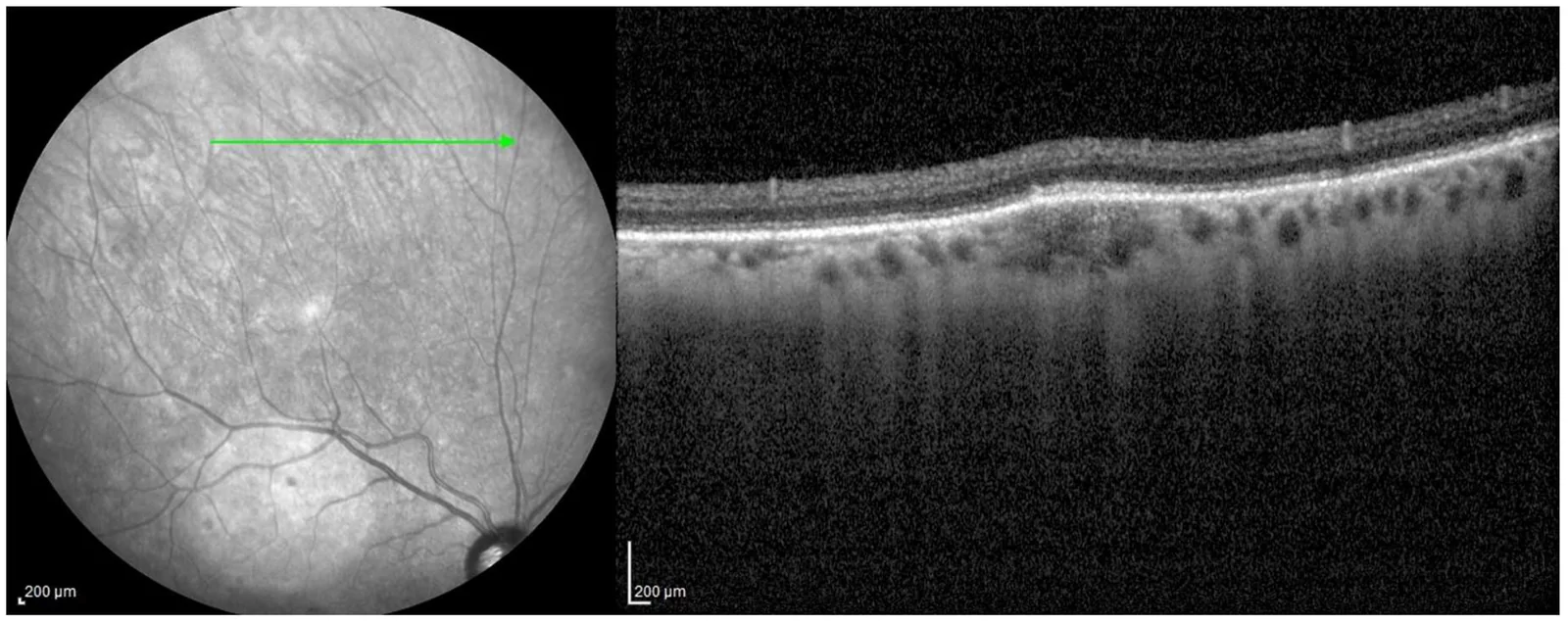
EDI-OCT (SPECTRALIS, Heidelberg Engineering GmbH, Heidelberg, Germany; software version 7.0.7) scan through a peripheral lesion in the superior retinal quadrant, showing focal choroidal infiltration with overlying photoreceptor disruption and increased backscattering.

The clinical course was further complicated by persistent photophobia, prompting comprehensive ophthalmologic evaluation with multimodal posterior segment imaging. Fundus examination revealed multiple ill-defined whitish-yellow chorioretinal lesions involving the posterior pole and mid-peripheral retina of both eyes. Macular optical coherence tomography (OCT) demonstrated focal photoreceptor disruption associated with localized choroidal thickening and increased backscattering, predominantly temporal to the fovea in the left eye and superotemporal to the vascular arcades in the right eye. OCT angiography disclosed multiple choroidal flow-void areas corresponding to sites of structural abnormality, providing additional characterization of the choroidal involvement (Figure 1 and Figure 2).

Accordingly, in the absence of direct ocular microbiological or histopathological confirmation, the ocular findings were considered presumptive cryptococcal chorioretinitis in the setting of microbiologically confirmed CNS and bloodstream cryptococcosis.

Serial ophthalmologic follow-up was performed using fundus photography, OCT, and OCT angiography. Fluorescein angiography and indocyanine green angiography were initially deferred because of the patient’s clinical condition and limited cooperation. Reassessment performed ten days later demonstrated an early response to systemic antifungal therapy, characterized by a reduction in both the number and size of the chorioretinal lesions, normalization of choroidal architecture, and the development of early cicatricial changes, particularly involving the temporal perifoveal lesion in the left eye. Subsequent evaluation confirmed further regression of the retinochoroidal lesions and stabilization of residual scarring.

Best-corrected visual acuity remained preserved throughout follow-up (20/20 in both eyes), with no evidence of clinically significant visual impairment.

Antiretroviral therapy was temporarily withheld during the initial phase of antifungal treatment to minimize the risk of immune reconstitution inflammatory syndrome (IRIS) and was subsequently reintroduced after 28 days of antifungal therapy. Genotypic resistance testing showed no HIV resistance-associated mutations.

Progressive clinical improvement was observed, with resolution of fever, headache, and constitutional symptoms, accompanied by normalization of inflammatory and laboratory parameters. The patient was discharged in stable condition with continuation of high-dose fluconazole consolidation therapy and scheduled multidisciplinary outpatient follow-up.

## 3. Focused Literature Review and Discussion

### 3.1. Materials and Methods

A focused PubMed literature search was conducted for the period January 2016–March 2026 and last updated in July 2026, using MeSH and free-text terms combining cryptococcosis, ocular involvement, and HIV infection. The final search was performed in July 2026. The core search strategy was (cryptococcosis OR “Cryptococcus neoformans” OR cryptococcal) AND (ocular OR choroiditis OR chorioretinitis OR retinitis OR endophthalmitis OR uveitis) AND (HIV OR AIDS). Only reports involving adults (≥18 years) living with HIV were considered eligible. Publications outside these eligibility criteria were used, when appropriate, only to provide contextual information regarding specific pathophysiological or neuro-ophthalmological aspects and were not included among the selected cases.

### 3.2. Ocular and Neuro-Ophthalmological Manifestations of Cryptococcal Meningitis

In the Ugandan ASTRO-CM cohort, 184 of 696 adults (26.4%) with HIV-associated cryptococcal meningitis exhibited at least one external ophthalmic sign or symptom during the study period. However, this composite figure included manifestations of systemic disease, neurological abnormalities, and ocular findings. Specifically, 12.9% of participants had ophthalmic manifestations of systemic disease, 9.1% had ocular signs or symptoms, and 7.0% developed cranial nerve palsies involving nerves III, IV, VI, or VII. Subjective visual disturbance in the absence of other objective findings was reported in 4.6% of participants [13].

These findings demonstrate that ophthalmic manifestations of cryptococcal meningitis include neurological abnormalities, ocular motor disturbances, and localized ocular findings and should not be interpreted collectively as evidence of direct intraocular fungal invasion.

Visual impairment during cryptococcal meningitis is clinically relevant because multiple pathophysiological mechanisms may contribute to its development. Molloy et al. demonstrated that both elevated CSF opening pressure and high baseline fungal burden were independently associated with severe visual loss in HIV-associated cryptococcal meningitis: a CSF opening pressure ≥40 cm H_2_O was associated with greater odds of severe visual loss compared with an opening pressure <25 cm H_2_O, while a baseline fungal burden >6.0 log_10_ CFU/mL was independently associated with severe visual impairment [5]. These findings suggest that visual dysfunction may reflect the severity of CNS cryptococcal infection and intracranial complications rather than direct intraocular dissemination.

These data demonstrate that visual dysfunction may reflect the severity of CNS cryptococcal infection and intracranial complications rather than direct intraocular dissemination.

Different mechanisms of visual loss have also been proposed according to the timing and clinical presentation. Direct optic nerve involvement, optic neuritis, and optochiasmatic arachnoiditis have been proposed in rapidly developing visual loss, whereas more slowly progressive impairment has been associated with increased intracranial pressure [14].

Optic nerve compartment syndrome has additionally been proposed as a mechanism related to impaired communication between the intracranial and peri-optic subarachnoid spaces [4].

Papilledema should likewise be interpreted primarily in relation to intracranial pressure rather than as evidence of direct ocular fungal infection. Conversely, the absence of papilledema does not reliably exclude raised intracranial pressure [15].

These observations have direct infectious disease implications: ophthalmological examination may identify consequences of CNS disease but cannot replace direct evaluation and management of CSF opening pressure.

### 3.3. Retinal Abnormalities During Cryptococcal Meningitis

Ellis et al. [16] further illustrated the difficulty of attributing retinal abnormalities directly to intraocular cryptococcal infection. The study compared 35 adults with advanced HIV disease and cryptococcal meningitis with 35 HIV-positive controls without meningitis, showing retinal abnormalities in 79% of patients with cryptococcal meningitis examined on the first day compared with 17% of controls. Retinal whitening was the most frequently observed abnormality, followed by optic disc swelling, retinal hemorrhage, and vascular abnormalities. Importantly, however, the study was designed to characterize retinal abnormalities associated with cryptococcal meningitis and did not provide direct microbiological or histopathological confirmation of cryptococcal infection within the eye. Accordingly, although retinal abnormalities were considerably more frequent in patients with cryptococcal meningitis, their presence cannot be considered equivalent to proven intraocular cryptococcosis. Rather, these findings suggest that cryptococcal meningitis may be associated with a broad spectrum of retinal changes whose underlying mechanisms are heterogeneous and not necessarily related to direct fungal invasion of ocular tissues. This distinction is particularly relevant in advanced HIV disease, where infectious, vascular, inflammatory, and neurological abnormalities may coexist and contribute to the retinal phenotype [16].

### 3.4. Direct Intraocular Cryptococcosis: Clinical Manifestations, Routes of Dissemination, and Evidence of Ocular Infection

Direct intraocular cryptococcosis is less frequently documented and has been described as choroiditis, chorioretinitis, and, in more extensive presentations, endophthalmitis with retinal and vitreous involvement [6,8,9,10,11,12,17].

The literature consists predominantly of individual cases and small series, and the degree of etiological confirmation varies substantially.

The strongest evidence of direct ocular involvement derives from cases in which *Cryptococcus* was demonstrated within ocular tissue or intraocular fluid. Aderman et al. described a patient with advanced AIDS and disseminated cryptococcal infection who developed multifocal choroiditis. Following death and bilateral enucleation, histopathological evaluation identified encapsulated budding organisms consistent with *C. neoformans* within a choroidal lesion [8].

Heitor et al. [9] similarly reported a young woman with HIV and microbiologically confirmed cryptococcal meningitis who died shortly after hospitalization. Postmortem examination demonstrated disseminated cryptococcosis involving multiple organs, including both eyes. Histological examination showed numerous yeast forms compatible with *C. neoformans* within the choroid bilaterally.

These cases provide direct pathological evidence that the choroid can be involved as part of disseminated cryptococcosis.

Direct microbiological confirmation from intraocular fluid has also been described. Joseph et al. [10] reported an HIV-positive patient with progressive visual impairment and endogenous endophthalmitis associated with a subretinal abscess. Vitreous microbiological examination demonstrated *C. neoformans*, providing direct microbiological confirmation of intraocular disease. Concomitant cryptococcal meningitis or other CNS involvement was not reported in this case. This report therefore represents microbiologically confirmed intraocular cryptococcosis without documented concurrent CNS disease and illustrates that ocular cryptococcosis may present as severe endogenous endophthalmitis rather than isolated choroidal disease.

Hematogenous dissemination has been proposed as a mechanism by which *Cryptococcus* reaches the highly vascularized choroidal circulation [6,9].

Heitor et al. [9] describe infectious microemboli lodging within the choroidal circulation as a potential mechanism underlying multiple posterior-pole lesions.

Flores Herrera et al. [6] similarly discuss hematogenous dissemination and spread related to leptomeningeal disease as potential mechanisms of intraocular involvement.

In one of their reported cases, *C. neoformans* was recovered from several blood cultures and from CSF, while skin biopsy also demonstrated cryptococcal infection, documenting bloodstream, CNS, and cutaneous dissemination; vitreous culture was not performed, and direct intraocular infection therefore remained microbiologically unconfirmed despite the strong clinical plausibility of ocular cryptococcosis in the setting of disseminated disease [6].

The frequent bilateral or multifocal pattern reported in several contemporary cases [6,8,9,11,12] is compatible with a systemic route of dissemination; however, bilaterality alone is not specific for cryptococcosis and should not be regarded as proof of hematogenous seeding. The same limitation applies to the present patient, in whom cryptococcemia, CNS infection, and bilateral chorioretinal abnormalities provide a coherent clinical context for disseminated ocular involvement, although the route of spread and the ocular etiology remain inferential in the absence of direct ocular microbiological or histopathological confirmation.

Several reports describe multiple pale, creamy yellow or yellowish choroidal lesions involving the posterior segment.

Aderman et al. described discrete creamy-yellow lesions distributed throughout the posterior pole and noted that profound immunosuppression in AIDS may result in limited accompanying intraocular inflammation [8].

Larochelle et al. described multiple bilateral yellowish placoid choroidal lesions in a patient with disseminated *C. neoformans* infection. Visual acuity was preserved, and the authors explicitly defined the diagnosis as presumptive [11].

Sanfilippo et al. [12] similarly reported a patient with newly diagnosed HIV infection, cryptococcal meningitis, and disseminated pulmonary disease who had mild blurred vision but preserved 20/20 visual acuity; fundus examination showed findings compatible with HIV retinopathy together with bilateral multifocal choroidal lesions considered suggestive of cryptococcal choroiditis, which subsequently regressed during systemic antifungal therapy.

Flores Herrera et al. reported a more inflammatory phenotype in two patients with AIDS and previous *C. neoformans* meningitis. Their cases included chorioretinitis, uveitis, retinal vasculitis, papillitis, and vitritis [6].

The literature therefore describes substantial variability in both anatomical involvement and visual function.

Importantly, preserved visual acuity does not exclude structural choroidal disease, as illustrated by the present case, in which bilateral multifocal abnormalities were documented despite maintenance of 20/20 visual acuity throughout follow-up. This dissociation between anatomical involvement and visual function highlights the limitations of relying solely on ocular symptoms or visual acuity to identify intraocular involvement. Accordingly, routine dilated fundoscopic examination should be considered in patients with systemic or CNS cryptococcosis, particularly in those with advanced HIV disease and profound immunosuppression, to detect potentially subclinical posterior-segment abnormalities. However, the available literature remains insufficient to establish the sensitivity, optimal timing, or clinical outcome benefit of a universal cryptococcosis-specific ophthalmological screening strategy.

### 3.5. Diagnostic Certainty and Differential Diagnosis in Advanced HIV Disease

One of the most important methodological issues emerging from the literature is the heterogeneity of diagnostic certainty [6,8,9,10,11,12]. This concept is broadly consistent with the EORTC/MSGERC framework for invasive fungal disease, which distinguishes different levels of diagnostic certainty according to the available clinical and mycological evidence [18]. However, these consensus definitions are not specific to ocular cryptococcosis and were developed primarily for research purposes. Therefore, in the present review, ocular cases were categorized descriptively according to the actual method of confirmation: direct microbiological demonstration of *Cryptococcus* in intraocular fluid, histopathological demonstration in ocular tissue, or presumptive/clinically attributed ocular disease in the setting of microbiologically documented systemic or CNS cryptococcosis without direct ocular confirmation.

These categories are descriptive and should not be interpreted as a validated ocular-specific diagnostic classification.

The available evidence can therefore be described according to the actual method of confirmation:

- Ocular microbiological confirmation refers to cases in which *Cryptococcus* was demonstrated directly in intraocular fluid.
- Ocular histopathological confirmation refers to cases in which cryptococcal organisms were demonstrated in ocular tissue.
- Presumptive or clinically attributed ocular cryptococcosis refers to cases with microbiologically documented systemic or CNS cryptococcosis in which ocular disease was inferred from compatible ophthalmological findings without direct intraocular confirmation.

These terms describe the evidence available and should not be interpreted as a validated diagnostic classification. This distinction is particularly important for the present patient. *C. neoformans* was demonstrated in CSF and blood, but no intraocular specimen was obtained. The appropriate terminology is therefore presumptive cryptococcal chorioretinitis in the setting of disseminated cryptococcosis, rather than microbiologically proven ocular cryptococcosis.

The differential diagnosis of posterior-segment abnormalities in advanced HIV disease remains broad.

Aderman et al. [8] note that infectious choroiditis in AIDS has also been associated with organisms including *Pneumocystis*, *Mycobacterium tuberculosis*, *Mycobacterium avium-intracellulare*, *Histoplasma capsulatum*, *Candida albicans*, *Aspergillus fumigatus*, and *Toxoplasma gondii*.

Consequently, a multifocal choroidal phenotype cannot by itself establish cryptococcal etiology.

CMV retinitis represents a particularly relevant competing diagnosis in patients with profound HIV-associated immunosuppression [7], while ocular toxoplasmosis and tuberculosis should also be considered according to the clinical and epidemiological context [8]. The case reported by Canfield et al. [17] is particularly informative in this regard, as the patient had simultaneous cryptococcal meningoencephalitis and toxoplasmic encephalitis together with bilateral choroidal abnormalities, and the authors explicitly acknowledged that intraocular cryptococcal–toxoplasma coinfection could not be definitively excluded. This case illustrates an important infectious-disease principle: identification of one opportunistic pathogen in advanced HIV disease should not preclude continued evaluation for additional infections when organ-specific abnormalities remain incompletely explained. Concurrent opportunistic infections may also involve separate anatomical sites; in the case reported by Heitor et al. [9], disseminated cryptococcosis was demonstrated at necropsy together with CMV disease and oropharyngeal candidiasis.

HIV-associated retinal disease may provide an additional diagnostic confounder. Sanfilippo et al. [12] distinguished retinal hemorrhages, Roth spots, and cotton-wool spots interpreted as HIV-associated retinopathy from separate multifocal choroidal abnormalities considered suggestive of cryptococcal involvement. Taken together, these observations reinforce the importance of careful etiological attribution and caution against assuming that every retinal or choroidal abnormality identified in a patient with cryptococcosis is necessarily caused by *Cryptococcus* [9,12,17].

In the present patient, microbiological investigations for *Toxoplasma gondii*, CMV, syphilis, and tuberculosis were negative. Nevertheless, the absence of direct intraocular sampling remains an important limitation to definitive etiological attribution.

### 3.6. Role of Ophthalmological Imaging

Ophthalmological imaging is useful for characterizing lesion morphology, localization, and longitudinal evolution but does not provide etiological proof of cryptococcal infection.

Fundus examination allows assessment of the distribution and morphology of clinically visible lesions, while multimodal imaging can provide additional information on retinal and choroidal involvement. Fluorescein angiography may characterize retinal vascular abnormalities and papillitis, whereas indocyanine green angiography can reveal choroidal lesions that may be less evident on conventional fundoscopy [6]. Structural OCT provides high-resolution characterization of retinal and choroidal abnormalities and has been used in several reported cases of cryptococcal choroiditis [6,8,11,12]. In particular, Aderman et al. [8] provided clinicopathological correlation between OCT abnormalities and histologically confirmed choroidal cryptococcosis.

OCT angiography may further characterize choroidal perfusion abnormalities without the need for intravenous contrast. In a previously reported case of cryptococcal choroiditis, Vu et al. demonstrated choriocapillaris flow-void areas corresponding to hypocyanescent lesions on indocyanine green angiography, with preservation of the superficial and deep retinal vascular plexuses [19]. However, the available evidence remains limited to isolated case reports, and no pathogen-specific OCT-A pattern for cryptococcal choroiditis has been established. In the present patient, OCT-A similarly demonstrated choroidal flow-void abnormalities corresponding to sites of structural disease and enabled serial non-invasive assessment. These findings should therefore be interpreted as complementary imaging features rather than as diagnostic evidence of cryptococcal granulomas.

The main clinical characteristics, level of ocular etiological confirmation, treatment, and outcomes of the reviewed HIV-associated cases are summarized in Table 2.

### 3.7. Treatment and Clinical Outcomes

The treatment of reported ocular cryptococcosis has generally been undertaken within the broader management of CNS or disseminated cryptococcal disease. Systemic amphotericin B-based induction followed by azole therapy has been associated with regression of ocular lesions in several reported cases [6,12]. Sanfilippo et al. [12] documented gradual regression of bilateral choroidal lesions following treatment with amphotericin B and flucytosine followed by fluconazole, while Flores Herrera et al. [6] similarly reported improvement of chorioretinal lesions and associated inflammatory findings during systemic antifungal therapy. In more severe forms of intraocular disease, particularly endogenous endophthalmitis, adjunctive local therapy has also been used. Joseph et al. [10] administered intravitreal amphotericin B after microbiological confirmation of *C. neoformans* from vitreous fluid, although the anatomical and functional outcome remained poor, whereas Canfield et al. [17] reported adjunctive intravitreal amphotericin B in a patient with presumed cryptococcal choroiditis and concomitant toxoplasmic CNS disease.

The available literature remains insufficient to determine which patients with cryptococcal choroiditis benefit from adjunctive intravitreal therapy, as published reports are heterogeneous in ocular phenotype, disease severity, systemic involvement, level of microbiological confirmation, and therapeutic approach [6,10,12,17]. Accordingly, current evidence does not support a standardized ocular-specific antifungal regimen, and management should primarily be guided by the underlying cryptococcal syndrome and the severity of intraocular involvement, with ophthalmological assessment informing individualized decisions regarding local treatment. In the present patient, systemic antifungal therapy alone was associated with progressive regression of the ocular lesions and preservation of visual acuity; however, this temporal association does not establish that systemic treatment specifically prevented visual loss.

### 3.8. Position of the Present Case Within the Published Literature

The present case shares several features with previously reported HIV-associated cases: profound cellular immunosuppression, concomitant cryptococcal meningitis, evidence of disseminated infection, multifocal bilateral choroidal or chorioretinal lesions, and anatomical improvement during antifungal therapy.

Its diagnostic certainty differs from histologically or microbiologically proven intraocular disease because no ocular specimen was obtained.

The case therefore belongs to the group of presumptive ocular cryptococcosis associated with microbiologically proven systemic/CNS infection, similar in level of confirmation to several contemporary reports.

An important clinical observation is the persistence of normal best-corrected visual acuity despite bilateral multifocal disease. Similar dissociation between anatomical involvement and preserved visual function has been described in the cases reported by Larochelle and Sanfilippo [11,12].

The present case also provides longitudinal structural OCT and OCT-A documentation during systemic treatment. These imaging findings expand the descriptive characterization of the lesions but should not be interpreted as pathogen-specific diagnostic biomarkers.

## 4. Conclusions

Ophthalmological abnormalities are well documented during HIV-associated cryptococcal meningitis, but they represent a heterogeneous spectrum and should not be considered synonymous with direct intraocular cryptococcosis. Visual loss, papilledema, and ocular motor abnormalities may reflect CNS disease, intracranial hypertension, and fungal burden, while retinal abnormalities may also occur in advanced HIV disease without demonstrated cryptococcal invasion.

Direct intraocular cryptococcosis is less frequently documented and includes choroiditis, chorioretinitis, and endophthalmitis. Importantly, the level of etiological confirmation varies substantially between published reports. Cases with microbiological or histopathological demonstration of Cryptococcus in ocular fluid or tissue should be distinguished from cases in which ocular involvement is inferred from microbiologically proven systemic disease and compatible ophthalmological findings.

This distinction is particularly relevant in advanced HIV disease because multiple opportunistic infections may coexist and because neither lesion morphology nor systemic cryptococcosis alone establishes the etiology of an ocular lesion.

The present case illustrates presumptive bilateral cryptococcal chorioretinitis occurring in the context of microbiologically documented cryptococcal meningitis and cryptococcemia. Despite multifocal bilateral structural disease, visual acuity remained preserved, while serial imaging documented progressive anatomical regression during systemic antifungal therapy.

Current evidence remains limited predominantly to case reports and small observational studies. Further studies using standardized ophthalmological assessment and clearly reported microbiological or histopathological evidence are needed to define the frequency, natural history, and clinical significance of direct ocular cryptococcal involvement in advanced HIV disease.

## Figures and Tables

**Table 1:** Serial cerebrospinal fluid findings during antifungal treatment.

Parameter	Day 0	Day 14	Day 21
Appearance	Clear	Clear	Clear
Color	Colorless	Colorless	Colorless
Serum albumin (g/dL)	3.2	2.8	3.3
CSF proteins (mg/dL)	102.8	161.3	42.6
Serum glucose (mg/dL)	96	90	90
CSF glucose (mg/dL)	24	24	48
CSF/serum glucose ratio (%)	25.0	26.7	53.3
CSF albumin (mg/dL)	63.5	82.3	20.4
Albumin quotient (%)	19.8	29.4	6.2
White Blood Cells (cells/ui)	128	18	10
Lymphocytes (%)	12	0	80
Monocytes (%)	23	95	10
Neutrophils (%)	65	5	10

**Table 2:** Clinical characteristics, ocular phenotype, level of etiological confirmation, treatment, and outcomes of published HIV-associated cases of ocular cryptococcosis included in the focused review. Diagnostic certainty categories are descriptive and do not represent a validated ocular-specific classification.

Study	HIV/Immune Status	Systemic/CNS Cryptococcosis	Ocular Phenotype	Direct Ocular Confirmation	Ocular Etiological Certainty	Ocular-Directed Treatment	Main Ocular/Clinical Outcome
Aderman et al., 2018 [8]	AIDS; CD4 7 cells/µL	Disseminated cryptococcosis with CNS involvement	Bilateral multifocal creamy-yellow choroidal lesions; focal choroidal thickening with outer retinal/RPE abnormalities on OCT	Yes—postmortem ocular histopathology demonstrated *Cryptococcus* within a corresponding choroidal lesion	Histopathologically confirmed	Bilateral intravitreal amphotericin B in addition to systemic antifungal therapy	Progressive systemic deterioration and death; postmortem examination provided clinicopathological correlation of the choroidal lesions
Joseph et al., 2018 [10]	HIV; CD4 105 cells/µL	Cryptococcal infection; No concomitant CNS cryptococcosis reported.	Unilateral endogenous endophthalmitis with subretinal abscess and severe visual impairment	Yes—vitreous microbiological examination positive for *C. neoformans*	Microbiologically confirmed	Vitrectomy and intravitreal amphotericin B in addition to systemic antifungal therapy	Poor visual and anatomical outcome despite treatment
Heitor et al., 2019 [9]	Newly diagnosed HIV/AIDS; 20-year-old woman	Cryptococcal meningitis; disseminated multiorgan cryptococcosis demonstrated at necropsy	Bilateral choroidal/chorioretinal involvement	Yes—postmortem ocular histopathology demonstrated cryptococcal organisms in the choroid	Histopathologically confirmed	No intravitreal treatment reported	Rapid clinical deterioration and death; bilateral ocular involvement demonstrated at necropsy
Canfield et al., 2020 [17]	AIDS; CD4 8 cells/µL	Cryptococcal meningoencephalitis with concomitant toxoplasmic encephalitis	Bilateral multifocal choroidal lesions	No	Presumptive; cryptococcal–toxoplasma ocular coinfection could not be excluded	Intravitreal liposomal amphotericin B in addition to systemic antifungal therapy; concomitant anti-*Toxoplasma* therapy	Clinical and visual improvement; definitive etiological attribution of the ocular lesions remained uncertain
Larochelle et al., 2020 [11]	HIV/AIDS; CD4 75 cells/µL	Cryptococcal meningitis with disseminated disease including cutaneous involvement	Bilateral multiple creamy-yellow/placoid choroidal lesions; clear vitreous	No	Explicitly presumptive	None; systemic antifungal therapy only	Visual acuity remained 20/20 OU; ocular lesions were not associated with significant visual impairment
Flores Herrera et al., 2021—Case 1 [6]	AIDS; CD4 61 cells/mm^3^	Recurrent cryptococcal meningitis; *C. neoformans* documented in blood and CSF; cutaneous cryptococcosis	Bilateral chorioretinal lesions with vitritis, retinal vasculitis and papillitis; macular involvement	No—vitreous culture not performed	Clinically attributed/presumptive	No intravitreal treatment reported; systemic antifungal therapy	Marked anatomical and inflammatory regression; visual improvement with residual macular scarring in the more severely affected eye
Flores Herrera et al., 2021—Case 2 [6]	Newly diagnosed HIV/AIDS; CD4 12 cells/mm^3^	*C. neoformans* documented in CSF and blood	Bilateral deep choroidal lesions with vitritis, papillitis, occlusive retinal vasculitis and focal serous retinal detachment	No—no direct ocular microbiological confirmation	Clinically attributed/presumptive	No intravitreal treatment reported; systemic antifungal therapy	Significant regression of funduscopic and multimodal imaging abnormalities during follow-up
Sanfilippo et al., 2024 [12]	Newly diagnosed HIV infection	Cryptococcal meningitis with pulmonary dissemination	Bilateral multifocal choroidal lesions associated with separate findings attributed to HIV retinopathy	No	Suggestive/clinically attributed	None; systemic antifungal therapy only	Progressive regression of choroidal lesions; visual acuity remained 20/20
Present case	HIV; CD4 12 cells/mm^3^	CSF and blood cultures positive for *C. neoformans*	Bilateral multifocal whitish-yellow chorioretinal lesions; focal photoreceptor disruption and choroidal abnormalities on OCT with corresponding OCT-A flow-void areas	No	Presumptive	None; systemic antifungal therapy only	Progressive anatomical regression during systemic therapy; BCVA remained 20/20 OU

Abbreviations: AIDS, acquired immunodeficiency syndrome; BCVA, best-corrected visual acuity; CD4, cluster of differentiation 4; CNS, central nervous system; CSF, cerebrospinal fluid; HIV, human immunodeficiency virus; OCT, optical coherence tomography; OCT-A, optical coherence tomography angiography; OU, *oculus uterque* (both eyes); RPE, retinal pigment epithelium.

## Data Availability

The clinical data supporting this report are contained within the article. Further information may be made available by the corresponding author in accordance with applicable privacy requirements.

## Abbreviations

The following abbreviations are used in this manuscript:

AIDS	acquired immunodeficiency syndrome
CDC	Centers for Disease Control and Prevention
CMV	cytomegalovirus
CNS CSF	central nervous system cerebrospinal fluid
HIV	human immunodeficiency virus
IRIS	immune reconstitution inflammatory syndrome
OCT	optical coherence tomography
